# Hypernephroma or Mesothelioma of the Kidney

**Published:** 1913-09

**Authors:** James Swain

**Affiliations:** Professor of Surgery in the University of Bristol; Surgeon to the Bristol Royal Infirmary.


### HYPERNEPHROMA OR MESOTHELIOMA OF THE KIDNEY.

BY

JAMES SWAIN, M.S., M.D. Lond., F.R.C.S.,

*Professor of Surgery in the University of Bristol; Surgeon to the Bristol Royal Infirmary.*

---

THE so-called hypernephroma of the kidney is the most common of the renal neoplasms, but until recent years it was confused with carcinoma or sarcoma of that organ. According to Wilson,[1] 78 per cent. of all tumours of the kidney removed belong to this class of new growth.

In 1883 Grawitz[2] made the suggestion that these tumours were derived from adrenal "rests," and to them he gave the name of *strumæ lipomatodes aberratæ renis*, but in honour of this distinguished observer others have referred to them as Grawitzian tumours.

Grawitz based his conclusions on the origin of these growths from displaced adrenal tissue on the fact that the cells resemble those of the suprarenal gland, both in their arrangements in nests and columns and because they contain fat, which is not found in renal epithelium, but is frequently present in the cells of the suprarenal bodies. Moreover, the tumours are found beneath the kidney capsule, and are themselves encapsuled, as in the case of adrenal rests in other parts.

These fragments of adrenal tissue or adrenal rests are found elsewhere than in the kidney, such as in the liver, mesentery, testis, and along the course of the spermatic and ovarian vessels; and Glynn[3] raises the pertinent question as to why adrenal rests of the kidney should develop into tumours so frequently, when they rarely do so if situated in other parts of the body

[1] *Ann. Surg.*, 1913, lvii. 522.

[2] *Virchow's Arch. f. path. Anat.*, 1883, xciii. 39.

[3] *Quart. J. Med.*, 1912, v. 157.

where they are more common, and further points out the well known fact that renal tumours are never associated with abnormal sexual development, as is commonly the case with growths arising in the suprarenal capsules.

Many others besides Glynn have doubted the correctness of the explanation of these tumours suggested by Grawitz, which has been accepted without question until the past few years, and Wilson[1] considers the theory has been effectively contradicted by Stoerk[2] on the following grounds: "(1) The Grawitzian tumours most frequently develop at the lower pole of the kidney, where adrenal rests are not found; (2) the so-called fat of the cells of the Grawitzian tumours is usually not fat, but a vacuolation related to the contents of the cells; (3) the Grawitzian tumour is a tumour of the renal cortex and not of the renal capsule, in which adrenal rests are usually found; and that (4) though the Grawitzian tumours do frequently contain cordons, which, however, only remotely resemble those found in the suprarenal, yet they almost invariably contain tubules, the analogues of which are never seen either in the normal suprarenal or in the tumours of that gland."

After a careful consideration of the embryological development of the kidney, Wilson shows good reason for his hypothesis "that these tumours are mesotheliomas, or more definitely nephromas, that is that they are elaborated from masses of nephrogenic tissue which have never become connected with the renal pelvis, and which have never attained adult type in either form or function."

If, however, it is admitted that the pathogenesis of these tumours is involved in some obscurity, there can be no difference of opinion as to their clinical importance and their essentially malignant character, though they are less rapidly fatal than the sarcomatous form of malignant disease of the kidney, which is more common in infancy or early childhood.

The symptoms are not characteristic, but resemble those found in other forms of malignant renal neoplasms. At first

[1] *Loc. cit.*

[2] *Beitr. z. path. Anat. u. allg. Path.*, 1908, xliii. 393, quoted by Wilson.

there is frequency of micturition, soon followed by pain in the back of an aching character, and reflected along the ilio-inguinal and ilio-hypogastric nerves. Sudden and profuse hæmaturia is said to be a common symptom, though I have not found it so; and as the renal substance is gradually absorbed, rather than invaded, by the growth of the tumour, and the renal pelvis is not necessarily involved, there seems to be no reason for expecting hæmaturia to occur; but should it take place it would probably be accompanied by some renal colic, owing to the presence of blood-clot in the ureter, though the pain in the back frequently alternates with the occurrence of bleeding. In this last respect the symptoms differ from the hæmaturia and pain which occur in association with renal calculus. The urine presents no special features. A tumour is found presenting the characteristics of a renal swelling, and later on metastases occur; the general health of the patient begins to decline, and the usual cachexia of malignant disease ushers in the final stage.

The primary growth may occur at either pole of the kidney, and increasing gradually in size, infiltrates the surrounding structures, extends along the veins, and may even invade the vena cava.

The secondary deposits have a special tendency to involve the long bones, lungs and liver, and as in the case of the thyroid gland, these metastatic deposits may occur at a time when the primary focus is small and unsuspected, so that it is always desirable to consider the possibility of the existence of a mesothelioma of the kidney when confronted with malignant disease of one or other of the long bones.

A slight febrile disturbance sometimes occurs during the progress of the disease, and a varicocele may form as in other cases of malignant disease, involving the left renal vein or vena cava.

The rate of growth of the tumour is comparatively slow, and when examined after removal it is found to have an irregularly rounded outline with the remains of the kidney substance stretched over it. On section it shows yellowish patches mixed with hemorrhagic areas, some of which show a tendency to cyst formation. Microscopically the cells are of a polymorphous epithelioid character arranged in nests or columns, but the supporting stroma runs between the individual cells. Histologically, therefore, the growth approaches the sarcomata in structure, in spite of the epithelial form of the parenchymatous cells.

The narration of the two following cases, which have recently been under my care, will serve to exemplify some of the foregoing statements :—

**Case 1.**—Mrs. G., æt. 51, after a few months of slight malaise, suddenly had a severe attack of abdominal pain and sickness, lasting about twenty-four hours, on February 25th, 1911. A month later a swelling was accidentally discovered on the left side of the abdomen ; there was some aching pain in the back and frequency of micturition. The tumour slowly increased in size ; there was no hæmaturia and no loss of flesh. She was well nourished and of a healthy appearance. The right kidney was unduly mobile, coming out completely from under the ribs. On the left side of the abdomen was a distinct prominence caused by a smooth, rounded, firm swelling, which filled the left flank behind and extended inwards as far as the mid-line. The swelling was almost entirely above the umbilical level, and the upper end passed under the costal margin. There was colonic resonance over the tumour, which came down freely on deep inspiration, and moved to the right of the mid-line when the patient was turned on her right side. A skiagram showed no sign of any calculus. The urine had a sp. gr. of 1020, was faintly acid ; urea, 2.3 per cent. ; no albumin or sugar ; a trace of indican, no casts, a few oxalate of lime crystals, no urates or uric acid, no red cells, and no bacteria of importance, 30 to 50 ounces per diem. On May 8th, 1911, I performed nephrectomy through the left semi-lunar line, the veins, which were tied before the kidney and tumour were removed, being an inch in diameter. The growth was about the size of a cocoanut, and occupied the upper pole of the kidney, the remains of the latter being about half the normal size, and situated at the lower end of the tumour. The tumour was more or less encapsuled, and on section was yellowish in some parts and dark red from breaking down hemorrhages in others. In less than a week the patient was passing 50 oz. of urine per diem, and made an uninterrupted recovery, gaining 13 lb. in weight before the end of the year.

In May, 1912—a year after the operation—there was no sign of recurrence, but she complained of occasional " lumbago "

pains; and in November, 1912, she wrote stating that she was "perfectly well." In the early part of this year (1913) she complained of some cough without expectoration, nausea, and occasional sickness, pain in the left side of the abdomen, but no "lumbago." On January 28th, 1913, I found no evidence of recurrence, but from this time her health began to decline, and she lost both flesh and strength. When I last saw her, on April 11th, 1913, there was a small, hard, fixed lump to be felt under the left costal margin, the liver was enlarged, but the spleen normal in size. She complained of a constant hacking cough and intercostal pain; the left lung was dull behind as high as the spine of the scapula, with diminished vocal resonance and fremitus, indicating a probable metastatic deposit in the lung and adjacent pleura.

On July 14th her medical attendant wrote: "The growth continues to spread and now reaches ensiform cartilage, but has not come much below the ribs; it seems to follow the diaphragm. Frequently her daily quantity of urine drops to about 12 ounces. She is then feverish and ill until it improves. Pain in chest is kept under by papine; morphia disagrees. When the right pleura is tapped it fills up again in about three days. I expect she will go on for some months longer. She is quite cheerful and well nourished, no wasting to notice. The liver has greatly increased in size, but there is little or no jaundice."

**Case 2.**—Mr. T., æt. 52, complained of aching pain in the right kidney region for about five months before I saw him. Though his appetite was poor, he did not think he had lost flesh. For some years he had been in the habit of getting up at night to pass water, and there was no increased frequency of micturition apart from this. During what he considered to be the duration of his present illness he had no hæmaturia, but he thinks he strained himself by lifting a heavy box two years previously, and this produced a painless hæmaturia for a week. His doctor found a large tumour in his abdomen, but the patient was unaware of its existence until then. He was distinctly anæmic in appearance, but fairly well nourished. Under the right costal margin and filling up the right loin was a large, firm tumour which reached downwards to one inch below the level of the umbilicus and inwards to one and half inches from the middle line. It moved downwards with respiration, had colonic resonance over it, and could be isolated from the liver above. There was a right-sided varicocele. An X-ray skiagram was negative. The urine was acid, sp. gr. 1022; some albumin, but no sugar, urates, blood or pus; urea 2.8 per cent. On examination, the blood showed 3,600,000 red corpuscles per c.m., hæmoglobin 50 per cent., colour index 0.68, leucocytes 16,250

per c.m., polymorphonuclears 74 per cent., large lymphocytes 17 per cent., small lymphocytes 7 per cent., eosinophiles 2 per cent.

On June 7th, 1912, I performed nephrectomy, tumour and kidney being removed through an incision in the right semilunar line. There was some rather sharp bleeding from the large veins of the pedicle, and the accompanying shock necessitated the injection of saline solution into the axilla. The tumour itself was about the size of a cocoanut and lobulated. On section the greater part was yellowish-white in colour, but there were some hemorrhagic patches. The kidney substance which remained was represented by a narrow area spread over the upper end of the tumour.

The patient made a good recovery, and went home about three weeks after operation. I heard no more of the patient until I wrote to his medical attendant, who informed me that the patient died in the October following operation "with secondary deposits in liver and abdomen."

There is no need to enlarge upon the details of these cases, as they are fairly typical of the disease; but it is worth noting how insignificant the symptoms may be until a comparatively late stage of the disease is reached.

The early phenomena may last for a considerable time—in some cases for several years—and it emphasises the importance of examining the kidney by palpation in all circumstances in which symptoms, however slight, may point to the possible existence of disease of that organ.

When a tumour is found there is generally little difficulty in saying that it is of renal origin, for a swelling filling the loin behind, growing forward with colonic resonance over it, and movable with respiration is almost certain to be some form of enlargement of the kidney, and should there be an associated hæmaturia the diagnosis practically admits of no doubt.

With careful observation there is little difficulty in excluding hydronephrosis, pyonephrosis, or cystic disease from these solid tumours; but the differential diagnosis of one form of solid tumour from another is largely conjectural. A rapidly-growing solid tumour of the kidney in an infant or young child is most probably a sarcoma; a slowly-growing solid tumour in the adult is highly suggestive of a hypernephroma.

The presence of a calculus can be excluded by skiagraphy, but there are some rare benign solid tumours (adenoma, fibroma, lipoma, etc.) of the kidney which, theoretically, might lead to an erroneous diagnosis. These benign tumours, however, constitute only 6 per cent. of all renal neoplasms, and therefore it can practically be said that all solid tumours of the kidney may be regarded as malignant.[1]

When the diagnosis is established the only rational treatment is by operation, provided that the tumour is not fixed, that there are no signs of metastasis in the long bones or other parts, and that the other kidney is healthy. Kidney and tumour must be removed by nephrectomy, preferably by the usual lumbar route; but where the tumour is large, as in the cases referred to above, the abdominal route must be chosen.

Nephrectomy for malignant disease of the kidney is a serious undertaking, but there is no alternative method of treatment that holds out the same possibility of cure. Unfortunately, the insidious character of the disease under discussion frequently allows the mischief to reach an advanced condition before it becomes manifest, and the patient sooner or later succumbs to the dissemination of the disease after operation.

The degree of malignancy varies in different cases of hypernephroma of the kidney, but the knowledge that this tumour is less rapid in its growth and dissemination than other varieties of malignant renal neoplasms should encourage us to exercise great vigilance, in order that we may not be deceived by the comparatively slight symptoms which occur in the earlier stages of this serious affection; for by a more prompt recognition of the disease we may hope to perform the necessary operation with a greater chance of success, both early and remote.

[1] Keen's *System of Surgery*, 1908, iv. 244.

